# Bites before and after bedtime can carry a high risk of human malaria infection

**DOI:** 10.1186/s12936-017-1740-0

**Published:** 2017-02-28

**Authors:** Masabho P. Milali, Maggy T. Sikulu-Lord, Nicodem J. Govella

**Affiliations:** 1 Ifakara Health Institute, Environmental Health and Ecological Sciences Thematic Group, Coordination Office, PO Box 78373, Kiko Avenue, Mikocheni, Dar es Salaam United Republic of Tanzania; 2 Marquette University, Department of Mathematics, Statistics and Computer Sciences, Milwaukee, WI USA; 3 QIMR Berghofer Medical Research Institute, Herston, QLD 4006 Australia

**Keywords:** Malaria, Mosquito, Parity, Biting time, Transmission, Mosquito age, Distribution

## Abstract

**Background:**

Understanding biting distribution of potentially infectious (parous) mosquitoes at various hours of the night would be useful in establishing the likely impact of bed nets on malaria transmission. Bed nets are highly effective at preventing biting by older malaria vectors, which occurs when most people are in bed. However, this behaviour is likely to vary across ecological settings and among mosquito populations.

**Methods:**

Field experiments were conducted in Minepa village within Kilombero Valley. Two outdoor catching stations located approximately 50 m from each other were established for mosquito collection. On each experimental night, mosquitoes were collected using human landing catch (HLC) by a single adult male at each station from 18:00 to 07:00 h. To compare the distribution of mosquito biting and the composition of their age structure, mosquitoes were sorted and recorded according to the hour they were collected. A sub-sample of *Anopheles arabiensis* was dissected to determine their parity status. Insectary-reared *An. arabiensis* within the semi-field system (SFS) with known age were also released in the SFS (10 m × 20 m) and recaptured hourly using HLC to determine the effect of parity on biting distribution.

**Results:**

Overall, there was no statistical association between the parity status and the biting time of *An. arabiensis* either in the field or in the SFS (*P* ≥ 0.05). The wild and insectary-reared *An. arabiensis* were observed to exhibit different hourly biting patterns.

**Conclusion:**

The study has shown that mosquito biting time phenotype is not influenced by their parity status. These findings imply that the risk of human exposure to potentially infectious bites is equally distributed throughout the night, thus supplementary measures to protect people against bites in evening and morning are desirable.

## Background

Historically, human malaria infections in sub-Saharan Africa occur mainly during late hours of the night. This period coincides with the peak biting behaviour of the primary malaria vectors: *Anopheles gambiae* sensu lato and *Anopheles funestus* [1, 2]. The risk of human infection depends mainly on two main factors: the human biting rate (the frequency at which a human is exposed to mosquito bites) and the proportion of the biting mosquitoes that are infectious [3, 4]. Only anopheline mosquitoes that are at least 10 days old can be infectious [5] because of the lengthy period required by the parasite to develop inside the mosquito, which is described as the extrinsic incubation period. While young nulliparous *Anopheles* never become infectious, the parous female may do so. Consequently, the proportion of mosquitoes that are infectious is proportional to the age of that mosquito populations [6, 7]. Therefore, the risk of infection at any given time of the night is influenced by the biting behaviour of the parous female mosquitoes [6, 7]: those that have previously had a blood meal and laid eggs [8–11].

Previous studies on *An. gambiae* in Sierra Leone, *Anopheles punctulatus* in Papua New Guinea [12] and *Anopheles darlingi* in Brazil [9] have provided evidence to indicate that parous *Anopheles* prefer to feed later in the night than the nulliparous population. The proportion of *An. gambiae* population that were parous in Tanzania was also slightly higher during late night hours (22:00–02:00) than earlier in the night (18:00–22:00) [13]. This overlap between the peak biting time of parous mosquitoes and the sleeping pattern of humans could explain why insecticidal-treated nets (ITNs) have been effective in interrupting human malaria infection across sub-Saharan Africa [14–19].

More recently, it has been reported that a substantial change in species composition of malaria vectors [20, 21] and a shift in biting time [21–27] is associated with the widespread use of ITNs across Africa. For instance, in Kilombero Valley in Tanzania, *An. gambiae* sensu stricto, which historically has been the dominant malaria-transmitting species, has been virtually eliminated [21, 28]. It has also been reported that the biting behaviour of mosquitoes is increasingly occurring before bedtime and outdoors [21, 29]. If the shift to bite before bedtime coincides with the increase in the proportion of parous mosquitoes, then the risk of malaria infection will be predictably relatively higher in this specific time window of the night compared to other time points.

Despite high coverage with ITNs, the villages around the Kilombero River still experience high malaria transmission rates [28, 30]. However, the influence of age of the main malaria vector species on their biting behaviour among these villages remains unknown. It is evaluated here for the first time.

## Methods

### Study site: Minepa village in southeastern Tanzania

The field study was conducted in Minepa village (S 08°16.4974′; E 036°40.7640′) within the Kilombero River valley in the Ulanga district of southeastern Tanzania [31] where malaria transmission remains high despite high coverage with ITNs. Most people in this village are subsistence farmers. The annual rainfall is between 1200 and 1800 mm, and the daily temperature is between 20 and 33 °C [32]. Members of the *An. gambiae* s.l. (*An. gambiae* s.s., *An. arabiensis*) and *An. funestus* are the primary malaria mosquitoes. However, *An. arabiensis* and *An. funestus* are currently the dominant species [21, 28] because long and widespread use of ITNs [33, 34] has virtually crashed the population of *An. gambiae* s.s. [21, 28]. Recent observations indicates that both *An. arabiensis* and *An. funestus* in this valley present active biting behaviour even before bedtime (18:00–22:00) [21], particularly when most locals are still outdoors [29].

## Experimental design

### Field sampling and processing

This experiment was conducted for two rounds each comprising a total of ten sampling nights. The first round was conducted towards the end of the rainy season between 21 and 30 April, 2016, while the second round was during the dry season 23 August to 1 September, 2016. Two outdoor catching stations, each approximately 5 m outside houses within the sampling area, were randomly chosen and established for mosquito collection. Standard randomization techniques were used: from the centre of the village two directions to work through the village were chosen by spinning a pen on a flat surface and the tenth house from each direction was chosen. Two volunteers out of four were randomly chosen and each randomly assigned to each catching station. Once assigned to a particular station, a volunteer was allowed to choose a counterpart to form a pair so that they can make a night shift with one start collection of mosquitoes from the first half of the night (18:00–24:00) and another finishing the second half of the night (24:00–07:00). Each pair remained at a particular station for a period of ten consecutive nights of sampling while alternating in night shift after each experimental night. In the second round, the two pairs exchanged houses and sampling continued for another ten consecutive nights in similar fashion as above. Mosquito collection was done by human landing catch (HLC), where a single male adult volunteer collected mosquitoes that landed on his exposed legs with a mouth aspirator as previously described [35, 36]. Mosquito collection was conducted for 45 min per hour, from 18:00 to 07:00, allowing a 15-min break for rest and refreshment. To compare distribution of mosquito biting behaviour and age composition per time point, hourly collections were placed in separate labelled paper cups corresponding to capturing time.

Each morning, with the aid of a stereo-microscope, all catches were sorted and morphologically identified in the field. Only mosquitoes identified as *An. gambiae* complex or *An. funestus* [1, 37] were considered for follow up. All other mosquito species were identified, recorded and then discarded. Individual mosquitoes were dissected and identified as either pre-gravid, nulliparous or parous, as previously demonstrated [38]. These mosquitoes were then individually preserved in 1.5 ml Eppendorf tubes containing desiccated silica gel for subsequent testing using polymerase chain reaction (PCR) assay [39] which determines sibling species identity. The enzyme linked immunosorbent assay (ELISA) was applied to test for the presence of a circumsporozoite protein in heads and thoraces of these mosquitoes [40, 41]. The heads and thoraces were heated in ELISA lysate at 100 °C for 10 min to gate away from false positive ELISA [42].

### Mark release recapture experiments in the semi- field system

Insectary-reared female *An. arabiensis* collected from wild larvae in Lupiro village within the Kilombero Valley were used. Mosquitoes were reared in an insectary built within the Ifakara Health Institute’s large semi-field system (SFS), measuring 21 × 9.1 × 7.1 m located at Kining’ina village (8.11417 S, 36.67484 E). Details of the design of the SFS can be seen elsewhere [43, 44]. The larvae from the field were transferred to a basin containing clean tap water using micropipette to discriminate predators. The basins were 12-l volume each with 300 ml of water. The basins containing larvae were covered with netting material and placed in racks established in the insectary within the SFS. The larvae were fed on Tetramin fish food (Tetra, Melle, Germany); temperature and humidity were not controlled during larvae rearing to mimic field conditions. Pupae were aspirated from the basins using micropipette and placed into small bowls (10 cm diameter) containing water and then transferred inside cages measuring 36 × 39 cm, allowing adult mosquitoes to emerge. The newly emerged wild adult female *An. gambiae* s.l. were maintained on 10% glucose solution until they were 3–5 days old when they were blood fed using arm feeding. Standard operating procedure (SOP) of arm feeding was followed, where well-trained technicians fed mosquitoes inside cages for 15 min. Prior to feeding, technicians were screened for malaria by rapid diagnostic test (mRDT) (MAL-Pf^®^, ICT Diagnostics, Cape Town, South Africa, which detects histidine-rich protein II) available in the laboratory. Only malaria free-technicians were allowed to enter and feed mosquitoes in the insectary. Before feeding, technicians put on gloves to avoid mosquito bites around the fingers. This procedure received ethical approval following the fact that *An. arabiensis* strain in this setting had repeatedly failed to adapt to feeding upon animals, or membrane feeding from previous trials. It was approved based on the fact that mosquitoes which are reared in the insectary within the screened enclosures (SFS) are pathogen-free so that technicians and/or volunteers are not exposed to the risk of contracting diseases.

**Fig. 1:**
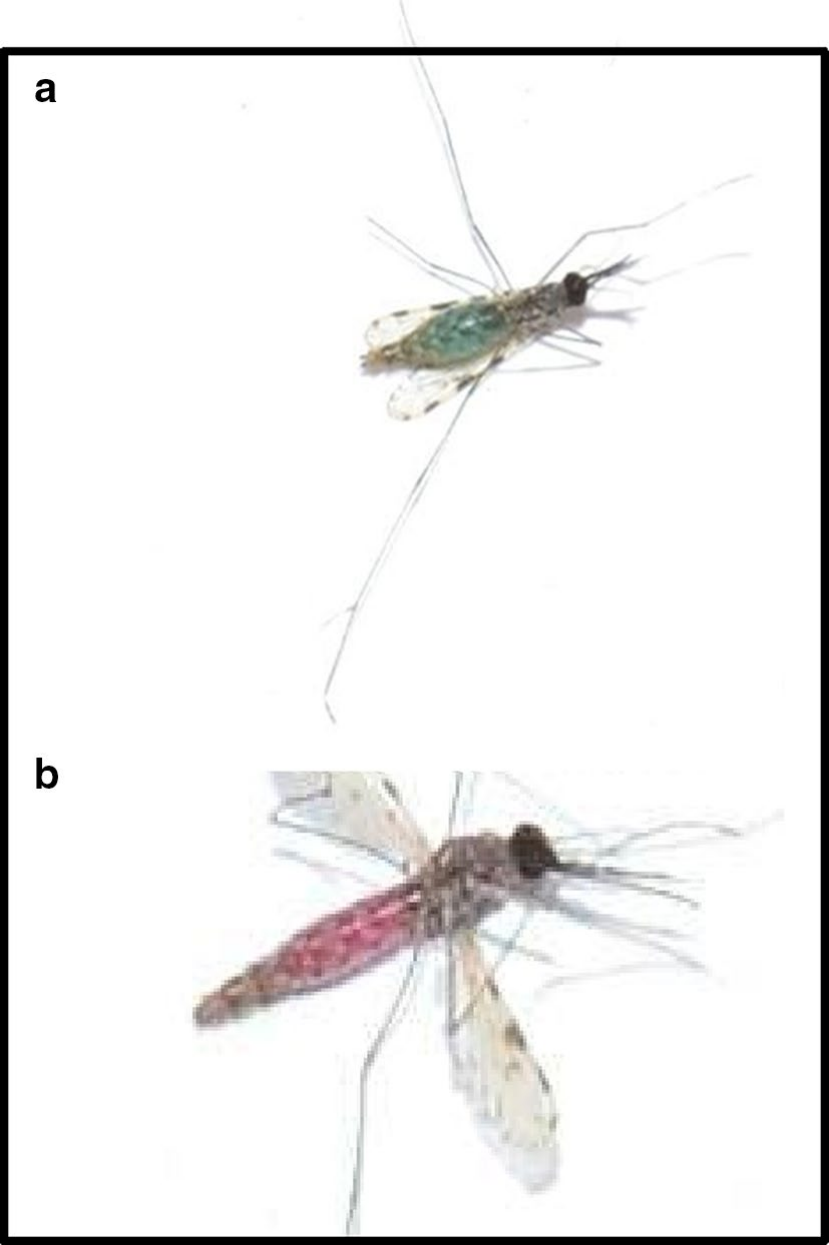
Images of an insectary-reared female *Anopheles arabiensis* in the semi-field. Fed on glucose solution containing 2 g/l synthetic* blue* food colour (**a**) or rhodamine B (**b**).* Blue* food colour was only visible in the abdomen (**a**), but rhodamine B was visible throughout in the thorax and abdomen

The fully fed female *An. gambiae* s.l. were individually placed into a correspondingly separate, labelled small cage (15 × 17 cm) with unique mosquito identification code (ID). Inside each small cage, a petri dish containing wet cotton lined on top with filter paper was provided, allowing mosquitoes to oviposit eggs. After oviposition, each individual was killed and stored in a correspondingly labelled 1.5-ml Eppendorf tube with desiccated silica gel and taken to central laboratory of the Ifakara Health Institute for confirmation of sibling species identification by PCR [39]. The newly emerged adult female F1 generation confirmed to be *An. arabiensis* only were placed into two separate cages each measuring 36 × 39 cm. In one cage, the young nulliparous group was maintained on 10% glucose solution alone, while in the second cage young mosquitoes were blood fed and allowed to lay eggs three times. Three to five days old nulliparous females and parous mosquitoes that had undergone three feeding cycles (at least 10 days old) were used. To discriminate parous from the nulliparous age group, one night prior to the release-recapture experiment, 200 nulliparous and 200 parous mosquitoes were placed in two different cages and were maintained on a mixture of 10% glucose with 2 g/l of either rhodamine B or synthetic blue food colour. The rhodamine B or blue food colour was assigned to either nulliparous or parous in randomized fashion using the lottery method. This randomization of markers between the two age groups was done after each experimental night. Although rhodamine B is a common biomarker for insects and was recently tested against sand flies [45], the use of synthetic food colour for marking mosquitoes is relatively rarely applied [46] compared to fluorescent dust dye [47, 48]. Nevertheless, this marking technique proved successful in this study (Fig. 1). The use of sugar-feeding dyes was preferred over fluorescent dust dye [47, 48] so that the two age groups could be distinguished from each other. Unlike sugar-feeding markers, fluorescent dust dye may contaminate the aspirator during recapturing and make it difficult to discriminate between the two groups. Even more importantly, these sugar-feed markers, particularly the rhodamine B, have been demonstrated not to affect longevity of the insects [45]. Mosquitoes were starved for at least 20 min before they were released. Mosquitoes were transferred and released in a separate chamber of the SFS containing natural vegetation, planted food crops and a small, thatched, mud-walled house designed to mimic the natural habitat of these mosquito species [44]. Mosquitoes were released from the centre of a chamber measuring 9.6 × 9.6 m at 17:00 by pulling strings held to mosquito netting cage. Both parous and nulliparous mosquitoes were released at the same time. A single adult staff was introduced into the chamber at 18:00 to perform HLCs from 18:00 to 07:00. Mosquitoes were recaptured at intervals of 1 h and placed in paper cups with a label corresponding to the time they were recaptured. Similar to field trials, recapturing was conducted for 45 min per hour with a 15-min break. This experiment was conducted for ten consecutive nights. HLCs were performed by two research staff alternating after each experimental night. Because disease-free, insectary-reared mosquitoes were used, no prophylaxis was given to the mosquito catchers [49]. Not all mosquitoes that were released per experimental night were successfully recaptured by HLCs. After each experimental night, in the morning two technicians carried out a thorough search for 30 min in the vegetation, hut, walls, and roof within the SFS and all mosquitoes that were found resting were collected, killed and discarded. Otherwise this would have affected the results, especially for the next experimental nights.

### Data analysis

Descriptive summary, tables and graphical analysis were used to examine the biting distribution across different times of the night for both wild *An. arabiensis* and *An. funestus* collected and insectary-reared *An. arabiensis*. Generalized linear mixed models (GLMM), using the R open source statistical software (version Rx 64 2.15.2) augmented with the *lme4* package, was applied to assess whether biting time phenotype was influenced or not by the parity status of mosquitoes and whether the proportion of parous varied between nights of sampling. The analysis of whether the proportion of parous recaptured varied between nights of sampling was only conducted with the SFS experiment. Very few pre-gravid mosquitoes were caught, and therefore they were combined with the nulliparous population and analysed as one group (nulliparous). Thus, the results of dissections were expressed as either nulliparous or parous. To test for the effect of parity on biting distribution across various times of the night for the field data, proportion of parous biting was treated as response variable with hour of the night first fit as a continuous variable and sampling night, rounds of collection and volunteers nested within station of collection treated as random effects. This allowed detection of whether there was any significant difference. This was followed by slight modification of the model where hour of the night at this stage was fit as fixed effect with random effects remaining the same as above, and model was run without an intercept. This was done so that absolute proportional of parous biting could be compared between each hour and also allow for plotting of graph fitted with 95% confidence interval. For the *An. arabiensis* reared in the SFS, testing the effect of time on parous biting distribution, the night of sampling and volunteer were treated as random effects with hour of the night as fixed effect and proportion of parous biting as response. Testing whether the proportion of parous biting varies over nights of experiment in the SFS, sampling nights were treated as fixed effect with volunteers as random effect and proportion of parous as response variable. It has been reported that differential attractiveness by mosquitoes to people does occur [50–52]. This could result in sampling variations over a certain time of the night or between sampling night, especially when mosquito capturing is performed by more than one person. It was also hypothesized that differential attractiveness by parous and nulliparous mosquitoes to people may exist, so volunteers were controlled in the model by treating them as random effects. Binomial distribution was used and the model fit were then separately plotted into graphical presentation. Very few *An. funestus* was caught and when tested with GLMM, spurious model fit was produced. Therefore, their number was considered too low to justify any robust statistical test, and the results associated with *An. funestus* were only reported descriptively.

## Results

A total of 5836 mosquitoes were caught over 20 nights of field collections. The catch included: 1710 (29.3%) *An. gambiae* s.l.; 211 (3.6%) *An. funestus*; 172 (2.9%) *Anopheles coustani*; 11 (0.2%) *Anopheles ziemanni*; 122 (2.1%) *Anopheles pharoensis*; 3610 (62%) *Culex* spp. (Table 1). A total of 1461 *An. gambiae* s.l. were successfully dissected. Of these, 63.2% (n = 924) were parous, 30.8% (n = 450) nulliparous and 6.0% (n = 87) pre-gravid. In the case of *An. funestus,* 200 specimens were successfully dissected. Of these, 66% (n = 132) were parous, 32.5% (n = 65) were nulliparous and 1.5% (n = 3) pre-gravid (Table 1). Overall, there were relatively more parous than nulliparous mosquitoes, a probable indicator of fewer mosquitoes emerging towards the end of the rainy season and during the dry season.

**Table 1:** Mosquito species, numbers collected and parity dissections of *Anopheles gambiae* sensu lato and *Anopheles funestus* from two rounds (21–30 April and 23 August-1 September, 2016) of data collection in Minepa Village, Kilombero Valley

Collection rounds	Total catch	Mean catch per night	Total dissected	Parous	Nulliparous	Pregravid	Parous (%)
Round 1							
*Anopheles gambiae* s.l.	724	72.4	631	371	187	73	58.8
*Anopheles funestus* s.l.	56	5.6	51	32	17	2	62.7
*Anopheles coustani*	13	1.3	N/A	N/A	N/A	N/A	N/A
*Anopheles ziemanni*	5	0.5	N/A	N/A	N/A	N/A	N/A
*Anopheles pharoensis*	9	0.9	N/A	N/A	N/A	N/A	N/A
*Culex* spp.	2301	230.1	N/A	N/A	N/A	N/A	N/A
Round 2							
*Anopheles gambiae* s.l.	986	98.6	830	553	263	14	66.6
*Anopheles funestus* s.l.	155	15.5	149	100	48	1	67.1
*Anopheles coustani*	159	15.9	N/A	N/A	N/A	N/A	N/A
*Anopheles ziemanni*	6	0.6	N/A	N/A	N/A	N/A	N/A
*Anopheles pharoensis*	113	11.3	N/A	N/A	N/A	N/A	N/A
*Culex* spp.	1309	130.9	N/A	N/A	N/A	N/A	N/A

Of the 4000 insectary-reared *An. arabiensis* within the SFS and released in the SFS, 1945 were recaptured. Of these, 69.4% (n = 1349) were parous and 30.6% (n = 596) were nulliparous. Of 1152 *An. gambiae* s.l. analysed by PCR, 96% (n = 1106) specimens were successfully amplified and all were identified as *An. arabiensis*. All identified *An. arabiensis* tested sporozoite negative. Contrary to that, two individuals (*An. funestus* s.s.) out of 181 from *An. funestus* s.l. were found sporozoite positive, with one biting between 21:00 and 22:00 and the other between 01:00 and 02:00. This implies that *An. funestus* s.s. might be more susceptible to infection than *An. arabiensis*. The *An. funestus* group was composed of *An. funestus* s.s 86% (n = 129), *Anopheles leesoni* 7% (n = 11), *Anopheles rivulorum* 4% (n = 6), and *Anopheles parensis* 3% (n = 4) of 150 successfully amplified specimens. Therefore, the field results presented here with respect to *An. gambiae* s.l. and *An. funestus* s.l. effectively reflect *An. arabiensis* and *An. funestus* s.s.

**Fig. 2:**
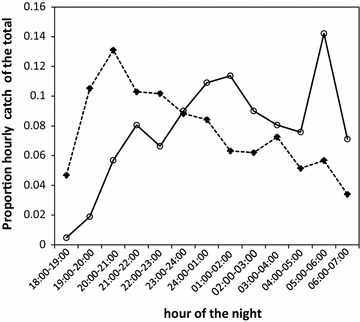
Distribution of biting times for wild *Anopheles arabiensis* and *Anopheles funestus* in Kilombero Valley, Tanzania. The *dashed line* represents *Anopheles arabiensis* and the *continuous line* represents *Anopheles funestus*

**Fig. 3:**
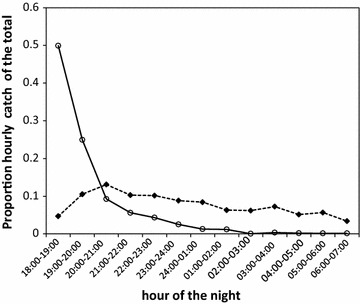
Biting activity of wild *Anopheles arabiensis* in the field compared with insectary-reared *Anopheles arabiensis* in the semi-field system. The *dashed line* represents the proportion of *An. arabiensis* that were captured biting at each hour of the night in the field and *continuous line* represents *An. arabiensis* that were recaptured biting at each hour of the night in the semi-field system

The two primary malaria vectors in the area, *An. arabiensis* and *An. funestus*, were observed to exhibit different biting activity over the course of the night. While the peak biting activity of *An. arabiensis* appeared to have started as early as between 20:00 and 21:00 and thereafter gradually decreased; *An. funestus* exhibited nocturnal biting behaviour with additional pronounced peak biting behaviour observed in the early morning (Fig. 2). Interestingly, the wild and the insectary-reared *An. arabiensis* within the SFS were also observed to exhibit different biting tendencies. Over 75% of all bites by the SFS-reared *An. arabiensis* occurred during the first 2 h of the early evening but drastically dropped for the rest of the night (Fig. 3).

**Fig. 4:**
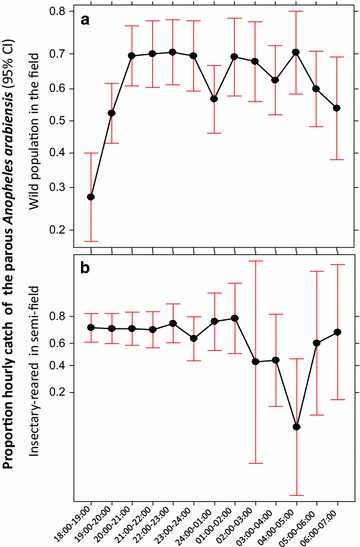
The proportion of parous *Anopheles arabiensis* that were sampled biting across different hours of the night. **a** Represents parous rate that were captured in the field; **b** represents those that were released and recaptured in the semi-field system. *Data points* represent absolute proportion of parous biting at each hour and *Bars* represent the 95% confidence interval. *X axis* represents hour of the night

**Fig. 5:**
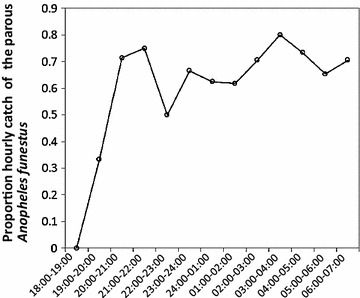
The proportion of hourly biting of the parous *Anopheles funestus*. *X axis* represent hour of the night

Table 2 summarizes hour by hour numbers of wild female *Anopheles arabiensis* and *An. funestus* caught and numbers of parous, nulliparous and pre-gravid. Generally, the numbers of parous biting mosquitoes appeared to be consistently higher relative to nulliparous (nulliparous and pre-gravid combined) across different times of the night, with the exception of the first hour of the evening (18:00–19:00), which was dominated by nulliparous mosquitoes for *An. arabiensis*. This observation is supported by the statistical test, which shows the lack of detectably statistical association between the parity status and the biting time of wild *An. arabiensis* from 19:00 throughout the night. The only statistical difference was detected when the catches between 18:00 and 19:00 were included in the model, where the proportion of parous catch was significantly less (z = 2.0, P = 0.045) (Fig. 4a). Although the numbers of *An. funestus* that were collected and dissected were too low to allow statistical test, proportion of parous biting from 20:00 appeared to be consistently ≥50% throughout the night (Table 2; Fig. 5). Similar to wild *An. arabiensis,* no apparent association was observed between biting time preference and parity status over the course of the night among the insectary-reared, released and recaptured *An. arabiensis* in the SFS. Only the biting activity between 04:00 and 05:00 appeared to be dominated by the nulliparous group (Fig. 4b).

**Table 2:** Hour-by-hour numbers of wild female parous and nulliparous (nulliparous and pre-gravid combined) of *Anopheles arabiensis* and *Anopheles funestus*

Hour	*Anopheles arabiensis*			*Anopheles funestus*		
	Parous	Nulliparous	Parous (%)	Parous	Nulliparous	Parous (%)
18:00–19:00	21	51	29.2	0	2	0
19:00–20:00	87	74	54.0	1	2	33.3
20:0–21:00	129	57	69.4	10	4	71.4
21:0–22:00	97	42	69.8	12	4	75
22:0–23:00	105	42	71.4	5	5	50
23:0–24:00	85	38	69.1	12	6	66.7
24:0–01:00	66	52	55.9	15	9	62.5
01:0–02:00	67	29	69.8	13	8	61.9
02:0–03:00	57	28	67.1	12	5	70.6
03:0–04:00	72	42	63.2	12	3	80
04:0–05:00	56	25	69.1	11	4	73.3
05:0–06:00	57	36	61.3	17	11	65.4
06:0–07:00	25	19	56.8	12	5	70.6

**Fig. 6:**
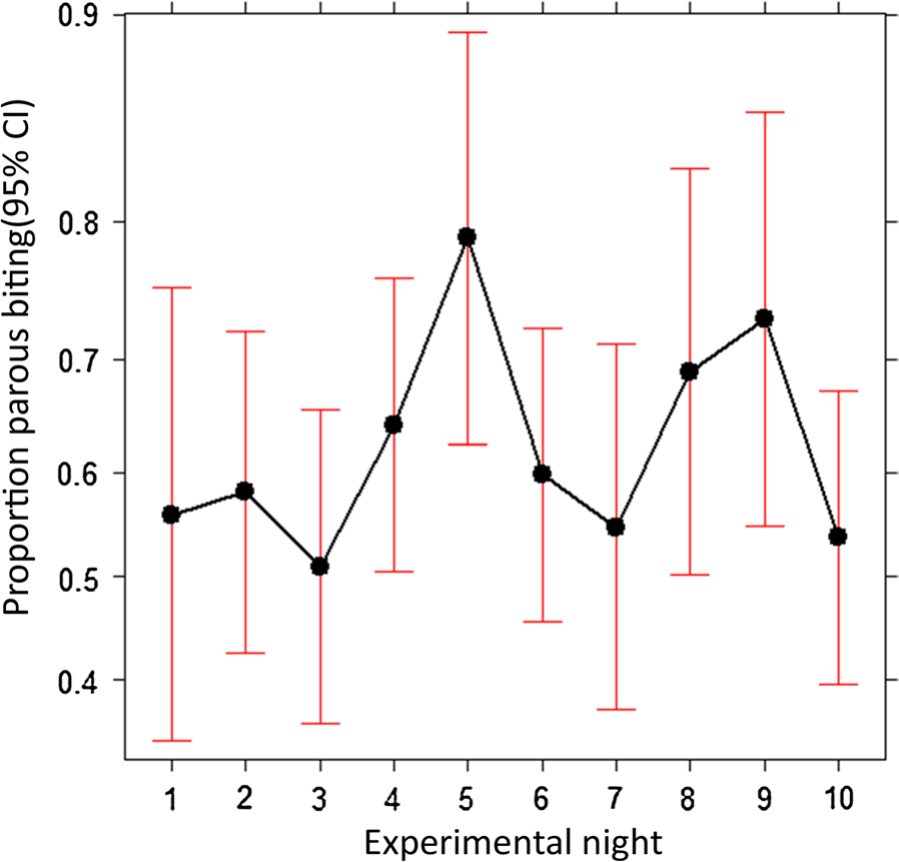
Distribution of the proportion of parous *Anopheles arabiensis* that were recaptured biting at each night of the study in the semi-field system. *Data points* represent absolute proportion of parous biting at each night of the study and *Bars* represent the 95% confidence interval. *X axis* represents night of the study

As shown in Fig. 6, the parous rate of insectary-reared, released and recaptured *An. arabiensis* within the SFS was consistently ≥50% throughout the ten sampling nights, with some fluctuation between nights. Statistical analysis indicated no evidence of significant variation in parity rates between all ten nights (Fig. 6).

## Discussion

A thorough understanding the biting behaviour of malaria vectors plays a crucial role in their control. Both *An. funestus* and *An. arabiensis* were collected, but the numbers of *An. funestus* were too sparse to be able to detect the existence of any statistical difference in their age structure distribution. This discussion will therefore focus mainly on *An. arabiensis*, the major vector of malaria in the Kilombero Valley, Tanzania, [21, 28, 29] and in other locations in sub-Saharan Africa [20, 24, 26, 27, 53]. The main objective was to assess whether there was an association between parity status of mosquitoes and their biting time in an area with widespread use of ITNs. Overall, the results indicate that parity status did not influence the biting time behaviour of *An. arabiensis* either under a full-field or a semi-field setting. A relatively higher proportion of parous wild *An. arabiensis* were observed to bite during the early hours of the evening but this proportion was not significantly different from other time periods of the night. These findings differ from previous reports on *An. gambiae* in Sierra Leone, *An. punctulatus* in Papua New Guinea and *An. darling* in Brazil, where the biting activity of the parous population predominantly peaked during bedtime, while the nulliparous population preferred to bite prior to or after bedtime [9, 12]. While local population variations in biting time behaviour with respect to mosquito age could possibly describe this difference, variation in the analytical approach may also matter. In previous studies, the proportion of parous was obtained through aggregating their numbers captured in ordered time intervals (e.g., 18:00–22:00) [12]. This may mask the effect occurring at each time-period falling within such time window. The findings from this study are however consistent with reports on *An. gambiae* and *An. funestus* in Burkina Faso [54] and *An. funestus* in Tanzania [11]. These findings also support the conclusive statement by Gillies that opposed the idea that age of *An. gambiae* was an important characteristic in determining biting time. Although in his study he found on average more young mosquitoes biting in the early part of the night than in the middle hours (22:00–02:00), these observations were found to vary as a function of weeks. For instance, the proportion of young mosquito biting was highest in the middle hours of the night in 4 weeks out of an 8-week study [13]. The absence of clustering of parous mosquitoes at specific time periods of the night may imply that the risk of human exposure to potentially infectious bites [6] is equally distributed throughout the biting window of these vectors. This also implies that protection against bites from these mosquitoes at all times is key to preventing malaria transmission. These suggestions may be supported by a recent epidemiological study in urban Dar es Salaam, Tanzania, which demonstrated that people who sleep inside houses with complete window screening and under a bed net enjoyed a reduction in infection risk only if their evenings and mornings were also spent indoors [55]. Time spent outdoors in the evenings and time of leaving the house in the mornings rather than living in a quality house alone [56], appeared to matter significantly in determining human infection risk [55].

Host-seeking *An. arabiensis* and *An. funestus* were observed actively feeding at times when most local people are usually outdoors engaged in different activities. Most outdoor activities in Valley of Kilombero occur on average before 22.00 and after 05.00 [29]. This overlap in time and space between mosquito and human activities increases the risk of human exposure to mosquito bites outdoors and consequently infection transmission [55]. This early evening biting peak of *An. arabiensis* is consistent with a previous report from the same valley [29]. It is also consistent with results from other parts of Tanzania [57] and beyond [22]. The early morning peak biting by *An. funestus* may be reported for the first time in this setting, however, similar observations have been reported elsewhere, including recent reports from West Africa [25, 58]. Early evening and morning outdoor exposure of humans to mosquito bites has epidemiological importance in terms of controlling transmission in this setting, and possibly across sub-Saharan Africa and beyond [24, 59, 60] where ITNs and/or indoor residual spraying (IRS) remain the only interventions. New interventions should focus on disrupting malaria transmission beyond bedtime hours, specifically before and immediately after bedtime. Interventions such as insecticide-treated clothing, topical and spatial repellents [61–63], and the application of ivermectin [64] should be trialled.

In the SFS, more parous were recaptured compared to nulliparous. Whether this implies parous mosquitoes to be more active and aggressive in feeding than nulliparous counterpart remains unclear. The few existing studies, for instance on *Aedes albopitus*, although demonstrating variations in feeding responsiveness between parous and nulliparous females, such responsiveness was found to vary with time, and so was time dependent [65]. There could be many factors that affect this. It may also be true that parity effect of feeding propensity is species-specific; more work is needed to confirm this. It is also not clear whether the sugar-feed colour marking approach affected the propensity of these mosquitoes to feed; the low recapture rate of 49% is unsurprising in the SFS which contains vegetation (Lwetoijera et al. unpublished).

The biting time phenotype outcome observed with wild population of *An. arabiensis* in the field and in semi-field-reared *An. arabiensis* in the SFS was assessed to see if it compared well and thus genotype. The two mosquito populations, although of the same taxon and originated from the same valley and subject to variable environments, exhibited different biting tendencies. *An. arabiensis* in the SFS was observed to respond to and started biting heavily immediately upon introduction of a human host in the semi-field in the evening and dropped to zero as the night progressed. To the contrary, the biting pattern of wild *An. arabiensis* in the full field was fairly distributed throughout the night (Fig. 3). This difference could clearly be due to the differences in environmental conditions they were exposed to. Although a search for a blood meal is mainly triggered by a physiological process in the mosquito, to locate, respond and successfully attack a host can be influenced by a number of environmental factors. These factors may include the distance between the host and the breeding site and the availability and accessibility of the host. Response to and successfully finding and attacking a host are the factors that are being measured in the field when characterizing behavioural phenotype outcomes of mosquito biting patterns [24, 25, 59, 66]. Therefore, the observed increasing early and outdoor biting behaviour by malaria vectors across sub-Saharan Africa and beyond [24, 25, 59, 66] may be described as driven mainly by phenotypic plasticity in response to variable environments, rather than to genetic change [67, 68].

This experimental design for addressing whether biting activity patterns observed in the field are influenced by genetic change or simply phenotypic plasticity of pre-existing behaviour had some limitations. Both field and SFS trials were supposed to be conducted in parallel on the same night and ideally with the SFS build at the vicinity of field collection so that the environmental variables between nights, such as temperature, humidity winds, moon light and cloud cover could also be controlled [69, 70]. Despite these limitations, this variation in behavioural outcomes observed between field and the SFS gives some insight into the role of availability and accessibility of host in determining biting time phenotypes but does not fully explain the observed field biting activity.

## Conclusion

The study has shown that mosquito biting time phenotype is not influenced by parity status. These findings imply that the risk of human exposure to potentially infectious bites is equally distributed throughout the night. The peak biting activity by *An. arabiensis* and *An. funestus* which is outside normal hours when people could access and use bed nets, calls for optimization of vector control approaches.
